# Calling for more Attention to Geriatric Suicidality in the Post-Pandemic Era

**DOI:** 10.14336/AD.2022.0409

**Published:** 2022-12-01

**Authors:** Che-Yin Lin, Yen-Kuang Lin, Li-Kai Huang, Yao-Tung Lee

**Affiliations:** ^1^Department of Psychiatry, Shuang Ho Hospital, Taipei Medical University, New Taipei city, Taiwan.; ^2^Graduate Institute of Athletics and Coaching Science, National Taiwan Sport University, Taiwan.; ^3^Center of dementia, Shuang Ho Hospital, Taipei Medical University, New Taipei city, Taiwan.; ^4^Department of Neurology, Shuang Ho Hospital, Taipei Medical University, New Taipei city, Taiwan.; ^5^Department of Psychiatry, School of Medicine, College of Medicine, Taipei Medical University, Taipei, Taiwan.

## To the editor,

COVID-19 is profoundly affecting life around the world. Simultaneously, the pandemic has led to psychological responses such as anxiety and depression. Suicide and self-harm are additional detrimental impacts, particularly in the elderly population [1]. As mutations of coronavirus are still threatening in countries worldwide, the long-term psychiatric sequelae of the COVID-19 and the consequent change in suicide rates have attracted growing attention. We highlight this issue by reporting two cases of elderly adults who developed symptoms of depression after the COVID-19 outbreak and subsequently attempted suicide after the peak of the pandemic. Written informed consent for publication of the clinical details was obtained from the patient.

Mr. H, a previously healthy and easy-going 72-year-old married male with no history of neuropsychiatric disorders presented to the emergency department (ED) following a suicide attempt by jumping off the bridge. He used to hike every day before the outbreak of the COVID-19, after which he was forced to stay at home for months. During the period, he began avoiding interactions with others after some conflicts with his family and withdrew from daily activities. These situations progressed even after the pandemic subsided remarkably in October 2021. In February 2022, he left his home without giving notice and was later sent to the ED by the police because of a suicide attempt. After evaluation by the geriatric psychiatrist, because of severe depression and strong suicide ideation, major depressive disorder was impressed and hospitalization for crisis intervention was suggested. However, Mr.H and his family refused and insistently applied against advice discharge because they regarded the hospital as a high-risk place for COVID-19 and feared the social stigma for depression.

Mrs. X, a previously healthy and cheerful 72-year-old married housewife with no history of neuropsychiatric disorders presented to the ED with frailty, hypotensive and extreme underweight. After the severe COVID-19 outbreak of local transmission in April 2021, she experienced excessive anxiety and insomnia. She was asked to remain in social isolation at home definitely by her children during which she became hypotalkative. In August, she began withdrawing from daily activities, refused to eat, and rested in bed for most of her time. These conditions persist even if the pandemic has slowed down in Taiwan. She was taken to a hospital where no organic cause relative to her symptoms was found after undergoing several medical examinations. Eventually, she visited the ED for frailty. After the evaluation by neurologist and psychiatrist, for the symptom of hopelessness and Cotard's syndrome, she was finally diagnosed of major depressive disorder with nihilistic delusion. She was subsequently admitted for treatment.

**Figure 1.**
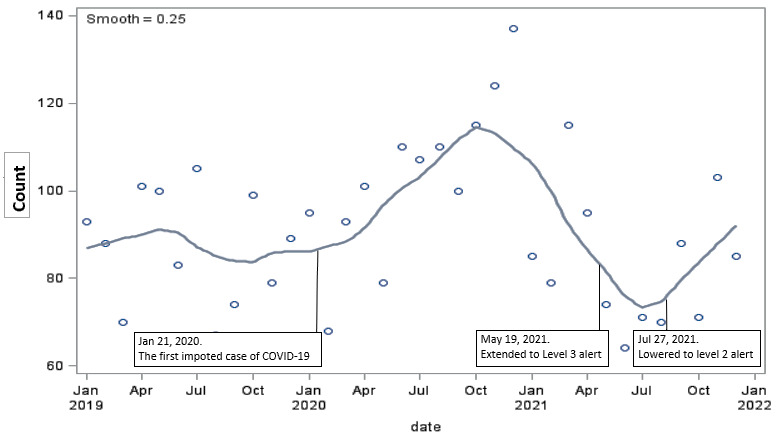
Number of suicide attempts in two of the most densely populated districts of New Taipei City, Taiwan.

Every 40 seconds, one person dies from suicide, making suicide a global public health challenge which shouldn’t be neglected in the pandemic. Although suicide numbers have remained largely unchanged or declined in the early months of the pandemic [2], Chen et al., recently reported that in elderly adults (≥65), the suicide rate decreased temporarily at the beginning followed by a sustained upward trend during the progress of the pandemic [3]. Our study revealed that the number of suicides may fall to lowest following the peak of the pandemic and afterwards rise again with decline of the infections (Fig. 1).

Social disconnection puts elderly at greater risk of depression [4]. Besides, the neuropsychiatric manifestations including depression may increase significantly in post-COVID-19 syndrome over time [5]. A possibility of delayed impacts for pandemic on suicide rates had been proposed [6]. In the period following a pandemic, the long-term mental influence of COVID-19 and the subsequent change in suicide rate especially among the elderly is noteworthy. Suicidality is considered a complex issue which integrate a process of bio-psycho-social and cultural interaction. Therefore, multidisciplinary, and multidimensional approaches across the different timelines should be considered for suicide prevention and intervention. In the post-pandemic era, we appeal that further research focus on this issue is needed and the corresponding policies such as disseminating accurate information and developing telehealth may be a matter of concern.
